# Miscellaneous ophthalmic conditions related to low CD4 cell count in HIV-positive patients

**DOI:** 10.22336/rjo.2024.38

**Published:** 2024

**Authors:** Mihaela Cobaschi, Anca Ruxandra Negru, Victor Daniel Dorobăț, Adrian Marinescu, Mioara-Laura Macovei, Cătălin Gabriel Apostolescu, Victoria Aramă

**Affiliations:** * “Carol Davila” University of Medicine and Pharmacy, Bucharest, Romania; ** “Prof. Dr. Matei Balş” National Institute for Infectious Diseases, Bucharest, Romania; *** Department of Infectious Diseases I, Faculty of Medicine, “Carol Davila” University of Medicine and Pharmacy, Bucharest, Romania; **** Department of Ophthalmology, “Carol Davila” University of Medicine and Pharmacy, Bucharest, Romania

**Keywords:** endophthalmitis, keratitis, neuroretinitis, HIV

## Abstract

**Introduction:** Management of patients living with Human Immunodeficiency Virus/Acquired Immunodeficiency Syndrome (AIDS) (PLWHA) still represents a challenge for doctors in various medical fields. The presence of co-infections, with different degrees of immune system impairment, raises the need for a multi-disciplinary approach to the PLWHA.

**Methods:** In this paper, we present three cases of PLWHA with various ophthalmological conditions, who were admitted to “Prof. Dr. Matei Balș” National Institute for Infectious Diseases (INBIMB). Three of them were late presenters, recently diagnosed with AIDS. All three were in immuno-virological failure. The ophthalmic conditions were either related to the HIV infection, or the result of other complications.

**Discussion:** The diversity and complexity of ocular involvement in PLWHA were deeply linked to the patient’s immunological status at the ophthalmological evaluation moment. Thus, antiretroviral therapy (ART) played an important immune status recovery role. Encountered ocular conditions vary, some being directly caused by the presence of the virus, and the others were the result of opportunistic infections (cytomegalovirus, Varicella virus) or other co-infections (Treponema pallidum). Neurological conditions disturbing the natural defense mechanism, prolonged hospital stay, and exposure to multiple antibiotic regimens are risk factors for difficult-to-treat eye infections with multidrug-resistant bacteria. Some ocular conditions can be the reason that leads to HIV infection diagnosis, while others can appear during the time, especially in patients with low ART adherence. The prognostic is conditioned by the early recognition and correct management of the disease and the immunological status recovery under ART.

**Conclusions:** Correct and early diagnosis of HIV-related eye conditions is mandatory to establish the most appropriate medical management to obtain an increase in the quality of life of the patient.

**Abbreviations:** HIV = Human Immunodeficiency Virus, AIDS = Acquired Immunodeficiency Syndrome, ART = Antiretroviral Therapy

## Introduction

Although the introduction of antiretroviral treatment has significantly increased the life expectancy and quality of life of patients living with HIV (PLWHA), their management remains a challenge for doctors and a multidisciplinary approach is always necessary.

In Romania, many patients are still diagnosed in advanced stages of HIV infection, especially in the years after the pandemic when access to the medical system was difficult. For this reason, these patients already have multiple opportunistic infections or other associated co-pathologies caused by the severely affected immunological status. In this context, a multidisciplinary approach to these cases is also mandatory to establish the correct management of the conditions of all patients and the most appropriate treatment for each [**1**]. It is well known that ocular conditions, especially when diagnosed in advanced stages and not treated correctly, are associated with irreversible impaired vision or even vision loss that leads to significant morbidity and low quality of life. Thus, PLWHA ophthalmologic evaluation, both at the time of diagnosis and during the disease, plays a role in the early detection of any conditions and in preventing permanent visual damage.

*Case 1*

A case of exogenous endophthalmitis with multidrug-resistant bacteria in the setting of multiple neurological impairments in a patient with an immune-virological failure due to low ART adherence and tuberculous meningoencephalitis, spondylodiscitis, and orchiepididymitis is presented.

It is the case of a 34-year-old patient, diagnosed with HIV infection from the age of 8, with low adherence to ART throughout the disease. In October 2022, he was admitted to „Prof. Dr. Matei Balş” National Institute for Infectious Diseases, Bucharest, Romania (INBIMB) with a diagnosis of tuberculous (TB) meningoencephalitis and TB spondylodiscitis, having a recent history of orchiectomy for TB orchiepididymitis.

Despite anti-TB treatment, multiple wide-spectrum antibiotic regimens administered for infectious complications developed over time, and initiation of ART according to current guidelines, the patient’s neurological evolution was unfavorable. He had multiple neurological complications even internal hydrocephalus, necessitating the placement of an external ventricular shunt. However, neurological deficits and consciousness continued to deteriorate even after the neurosurgical treatment. The patient developed left hemiplegia, thalamic spasticity, ocular globes deviation, and left eye lagophthalmos. Approximately two months post-admission, the patient exhibited purulent conjunctival secretions in the left eye, prompting an ophthalmologic opinion.

**Fig. 1:**
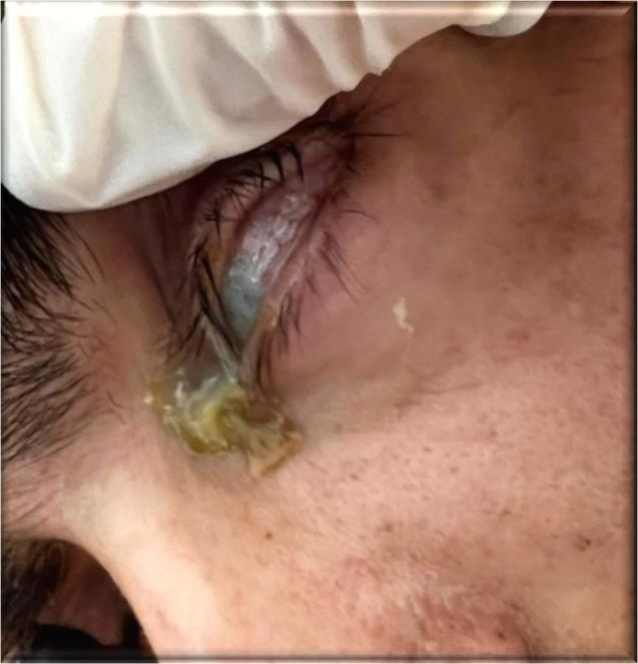
Ocular findings - day 1

Given the patient’s altered general condition, he could not be mobilized for an ophthalmologic exam in another clinic, not even the INBI’s ophthalmology office, so the examination was conducted at the patient’s bedside. The ophthalmologic examination revealed incomplete eyelid closure in the left eye, semi-dilated, non-reactive pupils in both eyes, greenish conjunctival secretions, marked conjunctival hyperemia, perikeratic injection, epithelial defect, and stromal infiltration in the lower third of the cornea, generalized corneal edema and hypopyon. All these findings were consistent with the diagnosis of exogenous endophthalmitis in the context of neurological impairment (**Fig. 1**). 

Initially, ceftazidime, gentamicin, and dexamethasone were administered subconjunctivally until conjunctival secretions culture results came positive with Pseudomonas aeruginosa, multidrug-resistant (MDR), sensitive to Colistin (Polymyxin E).

**Fig. 2:**
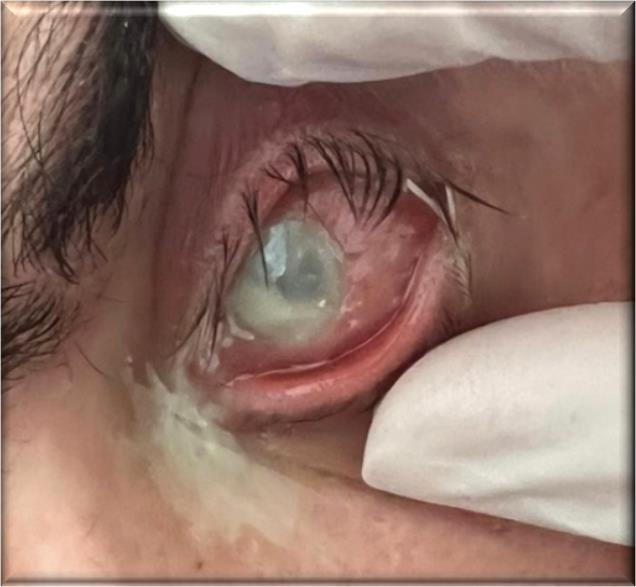
Ocular findings - day 2

Considering the patient’s inability to be transported to an ophthalmology unit, treatment was administered exclusively at the bedside (**Fig. 2**).

**Fig. 3:**
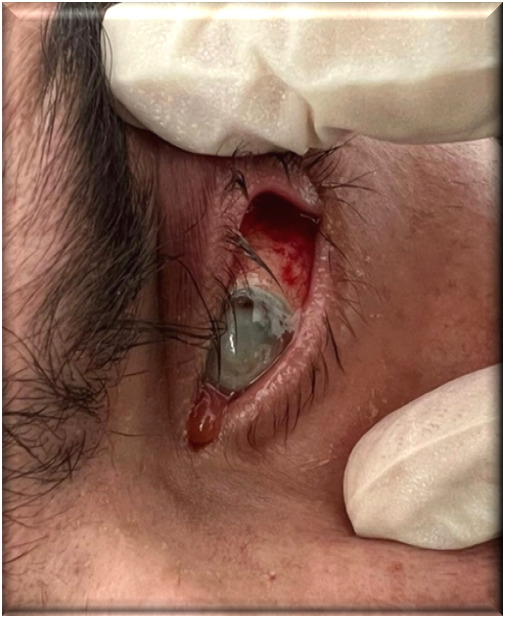
Ocular findings - day 4

Despite systemic treatment with Colistin and Meropenem for 21 days and topical ophthalmologic treatment with fortified colistin 0.19% (1 drop every hour), gentamicin sulfate (1 drop six times daily), ciprofloxacin (1 drop six times daily), voriconazole (1 drop six times daily), lubricating and epithelializing gel (1 application six times daily), and subconjunctival injections of Colistin and dexamethasone during the first three days, the progression remained unfavorable (**Fig. 3**).

**Fig. 4:**
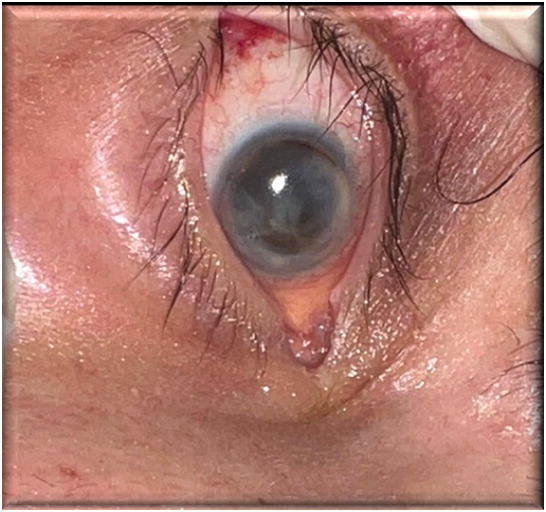
Ocular findings - day 14

Numerous imaging investigations were performed to assess the extent of the infectious lesion and diagnose the potential cerebral complications - cranial CT scans revealed no additional changes beyond those determined by TB. The patient’s extremely severe condition did not allow for transportation to an ophthalmology department for invasive and surgical treatment. Therefore, anti-infectious treatment continuation for an extended period was decided to prevent the spread of the infectious agent to cerebral structures. Approximately one week after systemic and topical treatment initiation, improvement was observed, and topical treatment was continued. After two weeks, a repeated culture from the conjunctival secretions was negative. At that time, extreme corneal thinning and imminent perforation were observed, so a therapeutic contact lens was applied, and topical antibiotic and antifungal treatment was maintained (**Fig. 4**). Over the following month, the patient’s general condition deteriorated, leading to death.

The particularity of the presented patient’s case was the difficulty of management, as he was non-transportable. In addition, all therapeutic measures initiated were limited by his deteriorated general condition.

*Case 2*

The second case presented is a case of Cytomegalovirus neuroretinitis in a newly HIV-diagnosed patient.

This is the case of a 30-year-old patient who presented with symptoms that began 10 days prior, including a nonproductive cough, fever, chills, and dyspnea. After testing positive for HIV, he was referred to INBIMB for acute respiratory failure. Upon admission, investigations revealed HIV RNA at 314,000 copies/mL and CD4 count at 12 cells/mm³, leading to a C3 HIV infection diagnosis.

The serology for CMV revealed negative IgM and positive IgG antibodies. He was also diagnosed with bronchopneumonia with methicillin-susceptible Staphylococcus aureus and Haemophilus influenzae, pneumocystosis being suspected. Systemic treatment was initiated with Ceftriaxone and Vancomycin for 15 days, oral Trimethoprim-Sulfamethoxazole for 21 days, and prednisone 40 mg twice daily, tapered afterward. Prophylaxis was provided for Mycobacterium avium complex (MAC), along with prophylaxis for thrombosis with Enoxaparin 0.3 mL/day. 

**Fig. 5:**
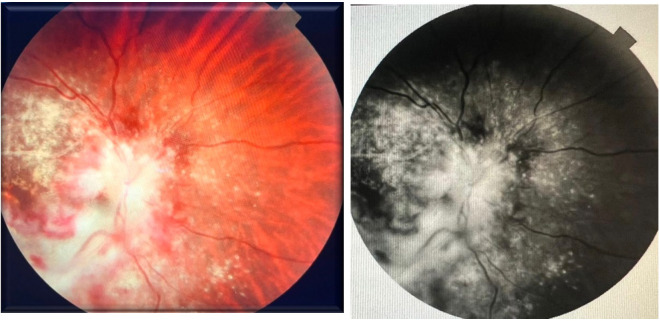
Cytomegalovirus neuroretinitis

During hospitalization, the patient reported sudden loss of vision leading to an ophthalmologic consultation. The ophthalmologic exam revealed a visual acuity of 20/20 in the right eye and no light perception in the left eye. The fundus examination of the right eye appeared normal, with no HIV-related pathological changes. However, the left eye showed an elevated, edematous optic disc with blurred retinal vessel emergence, peripapillary hemorrhages and exudates extending into the interpapilomacular space and macular area, diffusely dilated retinal vessels, and an occluded cilioretinal artery, findings suggestive of CMV neuroretinitis (**Fig. 5**).

Systemic oral treatment was initiated with valganciclovir 900 mg b.i.d. for 21 days, followed by 900 mg daily until the CD4 count exceeded 100 cells/mm³ for 3-6 months. ART with Triumeq (ABC/3TC/DTG) was also initiated, which was well tolerated. At the viro-immunological evaluation 4 weeks after starting ART, the CD4 count increased to 139 cells/mm³ and, given the significant ocular involvement and in concordance with the actual guidelines, it was decided to continue oral valganciclovir 900 mg daily for 3 months. The patient returned for an ophthalmological evaluation after 3 months, and the fundus appearance was stable without any additional complications. The patient did not recover vision but remained under long-term follow-up.

The particularity of the presented case referred to the sudden onset and complete loss of vision in one eye, with the contralateral eye not being affected. It is also noteworthy that the onset occurred directly through neuroretinal involvement in a newly diagnosed HIV-infected patient, with no possibility of visual recovery through systemic or local treatment.

*Case 3*

The third case presented is of an ocular involvement associated with coinfection of Treponema pallidum and Herpes Zoster in a newly diagnosed PLWHA patient.

We present the case of a 35-year-old man, recently diagnosed with HIV infection and referred to “Prof. Dr. Matei Balş” INBI for further investigations and ART initiation. The patient’s history included an episode of left thoracic zoster in 2021, for which he was evaluated and hospitalized in a provincial dermatology clinic, receiving antiviral treatment for 8 days. Subsequently, in September 2022, the patient presented to the same dermatology clinic with painful bilateral inguinal adenopathy, approximately 8/6 mm, mobile on superficial and deep planes. Clinical-biological evaluation and serology for Treponema pallidum were performed, with a positive result (VDRL/TPHA positive). He started a specific treatment regimen according to protocol, with 3 doses of benzathine penicillin IM, 2,400,000 units, and 3 doses at 7-day intervals. Meanwhile, vesicular lesions compatible with facial zoster appeared on the left eyelid and forehead, and the patient was then referred to INBIMB.

At admission, the patient presented with a moderately affected general condition, hemodynamically stable, afebrile, with marked bilateral conjunctival hyperemia and multiple vesicular lesions in the dermatome of the ophthalmic branch of the trigeminal nerve, violaceous lesions on the dorsal aspect of the lower limbs, chest, and upper limbs, well-demarcated violaceous plaques with a polycyclic contour located on the palate, a saburral and geographic tongue. The diagnosis of syphilis and herpes zoster ophthalmicus was established. The history of sexually transmitted diseases and repeated episodes of herpes zoster raised suspicion of HIV infection, which was confirmed by two positive serological tests and a positive Western Blot.

**Fig. 6:**
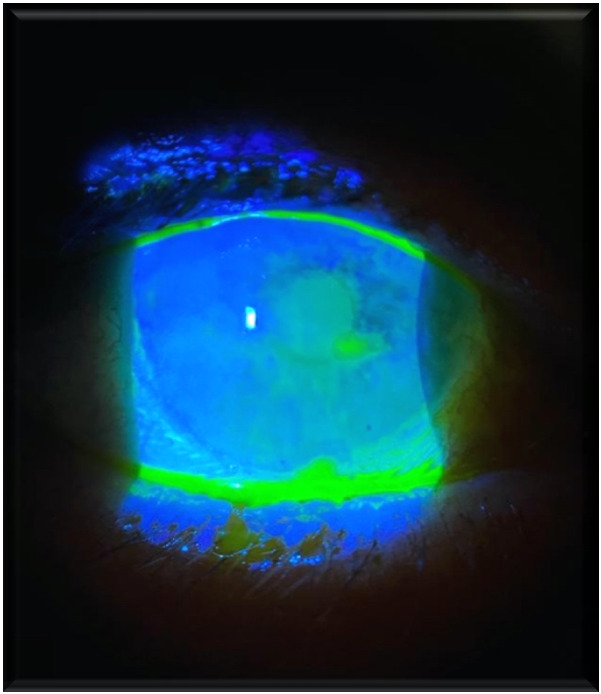
Zoster keratitis in HIV-infected patient

The ophthalmologic exam demanded in the setting of herpes zoster ophthalmicus diagnosis revealed vesicular lesions on the eyelid, marked hyperemia in the left eye, perikeratic injection, epithelial defect, and adjacent stromal inflammation (**Fig. 6**). Diffuse hyperemia in the right eye resolved with the instillation of phenylephrine.

Biological investigations revealed the following (**Table 1**): CD4 = 130 cells/mm³; HIV viral load = 171,000 copies/mL; viral markers for hepatic viruses: HBs antigen positive, anti-HCV antibodies negative, HBV viral load - 206,000 copies/mL, HDV IgM and IgG negative; syphilis serology - RPR: positive; VDRL: positive; CMV serology: IgG positive, IgM negative; Toxoplasma gondii serology: IgG positive, IgM negative.

**Table 1:** Initial evaluation and evaluation after one month of ART initiation

Parameter	Initial evaluation	One-month evaluation
Leukocytes	6530/mm³	6280/mm³
Neutrophils	56.5%	48.4%
Lymphocytes	35.2%	31.3%
Erythrocytes	5,210,000/mm³	5,110,000/mm³
Fibrinogen	3.25 g/L	1.87 g/L
CRP	1.28 mg/dL	0.15 mg/dL
Hemoglobin	15.3 g/dL	15.5 g/dL
HCT	43.9%	41.8%
ALT (TGP)	67 U/L	65 U/L
AST (TGO)	63 U/L	32 U/L
Urea	106 mg/dL	94 mg/dL
Creatinine	0.87 mg%	0.92 mg%
Cholesterol	214 mg/dL	225 mg/dL
HDL	38 mg/dL	33 mg/dL
LDL	78 mg/dL	85 mg/dL
Triglycerides	55 mg/dL	71 mg/dL

The established diagnosis was C3 stage HIV infection, HBV infection, recent latent syphilis, and herpes zoster ophthalmicus complicated by episcleritis of the right eye. Therefore, ARV treatment with Biktarvy (TAF/FTC/BIC) and antiviral treatment with acyclovir i.v. 10 mg/kg every 8 hours for 10 days were initiated. Ophthalmologically, treatment consisted of the following eye drops: for the right eye - non-steroidal anti-inflammatory drugs, ketorolac tromethamine, 1 drop x 3/day for 7 days, and artificial tears; for the left eye - ganciclovir ophthalmic gel 1 application x 5/day for 7 days, then 1 application x 3/day for 7 days, tobramycin 1 drop x 4/day for 10 days, and lubricating epithelializing gel 4 times a day for 10 days.

After 7 days of topical ophthalmic treatment, remission of episcleritis in the right eye and significant improvement of keratitis in the left eye were observed. Dermatologically, an improvement in the herpetic skin lesions was noted, with lesions in the crust stage at discharge. At discharge, the patient was recommended to continue ART treatment and Trimethoprim/Sulfamethoxazole for Pneumocystis prophylaxis until the CD4 count > 200 cells/mm³, being maintained for 3 consecutive months.

During follow-up, viro-immunological evaluation one month after the initiation of ART (**Table 2**) revealed an increase in CD4 to 258 cells/mm³ from 130 cells/mm³ and an HIV viral load of < 40 copies/mL from 171,000 copies/mL, indicating favorable evolution without adverse reactions to the recommended antiretroviral and antibiotic therapy (**Table 2**).

**Table 2:** Viro-immunological evolution

Parameter	Initial evaluation	One-month evaluation
CD4	130 cells/mm³	258 cells/mm³
HIV VL	171,000 copies/mL	<40 copies/mL

At the ophthalmologic evaluation one month after systemic and topical treatment initiation, complete remission of ophthalmic complications was noted. The prognosis was favorable, with the patient being adherent and compliant to antiretroviral therapy, achieving virological suppression one month after treatment initiation.

In patients diagnosed with a sexually transmitted disease, serological investigations should be extended to other diagnoses of infections that can be transmitted in this manner. In the presented patient, the diagnosis of secondary syphilis led to the diagnosis of HIV and HBV infections. Early detection and treatment of these infections are crucial. The particularity of the case presented was the simultaneous ophthalmologic involvement, the coexistence of episcleritis, and zoster keratitis with different management.

## Discussion

The presented cases involved three HIV-positive patients with various ocular conditions: Pseudomonas aeruginosa endophthalmitis, cytomegalovirus retinitis, herpes zoster ophthalmicus, and syphilis. Each infection presented itself as a unique diagnostic and therapeutic challenge, compounded by the patient’s compromised immune status due to HIV. The complexity of managing such co-infections highlighted the need for a nuanced and comprehensive approach.

First case

Pseudomonas aeruginosa is a common cause of opportunistic infection in HIV patients, particularly in those with neutropenia or hospitalized individuals. It can cause respiratory, urinary tract, and eye infections, and bacteremia [**2**]. Pseudomonas aeruginosa often exhibits multi-drug resistance, necessitating potent antibiotics such as ceftazidime, ciprofloxacin, piperacillin-tazobactam, and sometimes combination therapy [**3**].

Colistin remains a critical therapeutic option in the management of Pseudomonas aeruginosa endophthalmitis, especially in HIV patients in whom multidrug-resistant strains pose significant treatment challenges. This antibiotic’s efficacy against Gram-negative bacteria, including resistant strains, underscores its importance in achieving microbiological control and preventing vision-threatening complications [**4**].

Exogenous endophthalmitis caused by Pseudomonas aeruginosa in these HIV-positive patients with multiple and severe co-pathologies, with altered neurological status represents a significant clinical challenge due to the aggressive nature of the infection and the compromised immune status of the affected individuals [**5**]. Prompt diagnosis and aggressive treatment are critical to preserve vision and prevent systemic spread. Although the outcome of this patient was unfavorable, this case highlighted the importance of a multidisciplinary approach, combining infectious disease management with specialized ophthalmologic care, to optimize the patient’s outcomes. Early identification of pathogens through culture and sensitivity testing, along with tailored local and systemic antimicrobial therapy, is essential. Additionally, the role of ART in improving the overall immune function in PLWHA cannot be overstated, as it plays a crucial role in preventing such severe infections. 

Regular follow-up and comprehensive care are imperative to monitor for potential complications and to ensure the best possible prognosis for HIV-infected individuals suffering from severe ocular infections.

Second case

CMV retinitis is a leading cause of vision loss in patients with advanced HIV/AIDS. It leads to retinal necrosis and, if untreated, can result in blindness. Early diagnosis through fundoscopic examination is critical. Neuroretinitis caused by CMV in HIV patients with immune-virological failure is a severe and vision-threatening condition that underscores the complex interplay between opportunistic infections and immunosuppression. This case emphasized the critical need for early recognition and prompt initiation of antiviral therapy to prevent irreversible vision damage. Combined with effective antiretroviral therapy (ART), Valganciclovir has an important role in managing CMV neuroretinitis and improving immunological parameters [**6**]. Regular ophthalmologic assessments are essential for early detection and monitoring of treatment response. As demonstrated, comprehensive management, which includes systemic antiviral treatment and consistent ART, leads to significant immunological and ophthalmological improvement. Vigilance in monitoring for CMV-related complications and adherence to treatment protocols is paramount in improving outcomes for PLWHA. This case reinforced the necessity for integrated care approaches and highlighted the importance of maintaining high clinical suspicion for opportunistic infections in immunocompromised patients [**7**].

Third case

 Ocular syphilis can present with a range of manifestations. In the context of HIV, the progression of syphilis can be more aggressive, and ocular involvement can be severe. Ophthalmologically, ocular involvement in syphilis can be unilateral or bilateral and can manifest as conjunctivitis, keratitis, episcleritis, scleritis, neuroretinitis, chorioretinitis, papillitis, optic neuritis, or nerve palsies. Secondary syphilis is characterized by ring-shaped papules appearance. Lesions appear symmetric, erythematous, ring-shaped, and arcuate plaques, predominantly on the scalp, lower body, perioral, perianal, and genital regions. Ring-shaped lesions can vary in size and appearance. Both HIV-positive and HIV-negative patients receive the same treatment for syphilis. The treatment of choice for all stages of syphilis is long-acting penicillin [**8**].

Herpes zoster occurs in the general population in about 3.6 cases per 1,000 people, more frequently in older and immunocompromised individuals. Before antiretroviral therapy became accessible, HIV-infected patients had an incidence of herpes zoster more than 15 times higher. HIV-positive adults can develop herpes zoster at any CD4, but the risk increases at CD4 levels below 200 cells/mm³ [**9**,**10**]. Herpes zoster is characterized by a painful, erythematous maculopapular rash, followed by vesicle formation and subsequent crusting. The cutaneous lesions are associated with neuropathic pain in the affected dermatome [**11**]. Thoracic dermatomes are affected in 42.4% of herpes zoster cases, followed by cranial nerve dermatomes 28.2%, and cervical 12.1% [**12**]. One or more consecutive episodes of herpes zoster can affect the same dermatomes, necessitating HIV testing in cases of repeated herpes zoster episodes [**13**].

In HIV-positive patients, cutaneous lesions can extend over multiple dermatomes, with a risk of systemic infection (disseminated herpes zoster), especially in advanced stages of the disease with significant immunosuppression. Additionally, postherpetic neuralgia is more common in these patients [**14**].

The convergence of herpes zoster ophthalmicus and syphilis in HIV patients presents a challenging clinical scenario, requiring a multifaceted approach to diagnosis and management. This case underscored the significance of comprehensive serological testing and early intervention in HIV-positive individuals presenting with dermatological and ophthalmic manifestations. Prompt initiation of antiviral therapy for herpes zoster, coupled with appropriate antibiotic treatment for syphilis along with ATV initiation, is crucial in preventing complications and improving patient outcomes. Ophthalmological evaluation and treatment are essential to mitigate the risk of vision-threatening complications associated with herpes zoster ophthalmicus. Integrated care, involving infectious disease specialists, dermatologists, ophthalmologists, and HIV care teams, is mandatory for achieving optimal therapeutic outcomes and preventing disease progression. This case study emphasized the importance of a vigilant clinical approach and timely therapeutic interventions tailored to the specific needs of HIV-infected patients with concurrent infectious complications.

## Conclusion

The analysis of these exposed cases highlighted that the ocular pathology of the patients with HIV infection can be caused both by the viral infection itself, as well as by opportunistic infections or co-infections of the patients. All cases involved both ophthalmologic and systemic treatment, thus, a good collaboration between the infectious disease specialist, ophthalmologist, dermatologist, and neurologist was mandatory.

In conclusion, the care of HIV patients requires collaboration between multiple medical specialties, especially if the patients are in an advanced stage of HIV infection. A multidisciplinary approach to these patients provides the possibility of developing a correct plan to treat all the pathologies they suffer from.

**Conflict of Interest Statement**

The authors state no conflict of interest.

**Informed Consent and Human and Animal Rights Statement**

Informed consent has been obtained from all the individuals included in the study.

**Authorization for the use of human subjects**

Ethical approval: The research related to human use complies with all the relevant national regulations and institutional policies, as per the tenets of the Helsinki Declaration, and has been approved by the review board of “Prof. Dr. Matei Balş” National Institute for Infectious Diseases, Bucharest, Romania (C2786/14.03.2024).

**Acknowledgments**

None. 

**Sources of Funding**

None.

**Disclosures**

None.
